# Predictors of Outcome and Severity in Adult Filipino Patients with Febrile Neutropenia

**DOI:** 10.1155/2015/920838

**Published:** 2015-09-03

**Authors:** Marc Gregory Y. Yu, Ralph Elvi M. Villalobos, Ma. Jasmin Marinela C. Juan-Bartolome, Regina P. Berba

**Affiliations:** Department of Medicine, Philippine General Hospital, Taft Avenue, Ermita, 1000 Manila, Philippines

## Abstract

*Aim*. The study aimed to describe the profile of Filipino febrile neutropenia patients and to determine parameters associated with severe outcomes. *Methods*. This is a retrospective study of Filipino febrile neutropenia patients admitted to the Philippine General Hospital. Patients were described in terms of clinical presentation and stratified according to the presence or absence of severe outcomes. Prognostic factors were then identified using regression analysis. *Results*. 115 febrile episodes in 102 patients were identified. Regression analysis yielded prolonged fever >7 days prior to admission (OR 2.43; 95% CI, 0.77–7.74), isolation of a pathogen on cultures (OR 2.69; 95% CI, 1.04–6.98), and nadir absolute neutrophil count (ANC) < 100 during admission (OR 1.96; 95% CI, 0.75–5.12) as significant predictors of poor outcome. Factors that significantly correlated with better outcome were granulocyte colony-stimulating factor (G-CSF) use (OR 0.31; 95% CI, 0.11–0.85) and completeness of antibiotic therapy (OR 0.26; 95% CI, 0.10–0.67). *Conclusion*. Prolonged fever >7 days prior to admission, positive pathogen on cultures, and nadir ANC < 100 during admission predicted severe outcomes, whereas G-CSF use and complete antibiotic therapy were associated with better outcomes. These prognostic variables might be useful in identifying patients that need more intensive treatment and monitoring.

## 1. Introduction

Febrile neutropenia refers to the presentation of fever in a neutropenic patient, commonly with an uncontrolled neoplasm of the bone marrow, or in a patient undergoing cytotoxic treatment [1]. Around 50% of patients with solid tumors and 80% of those with hematologic malignancies will develop concomitant fever and neutropenia [2]; despite advances in diagnostics and therapy, these patients still face a mortality rate of 5–21.5% [3–5]. This is partly explained by the fact that neutropenic patients who develop fever have a 60% chance of being infected. Infections arise from defects caused by the underlying disease, those induced by cytotoxic drugs, and those associated with invasive procedures [6, 7]. With newer, more potent agents and dose-dense chemotherapy schedules, patients are faced with more severe and prolonged degrees of neutropenia leading to more complications and higher healthcare costs [8].

Gram negative organisms traditionally predominated as the most common causative pathogens in patients with febrile neutropenia [9, 10], although there is a rising incidence of Gram positive organisms among Western patients [11, 12]. In the Philippines, a retrospective study showed Gram negative bacilli (51.5%) as the most frequently isolated pathogen [3]; in another study involving pediatric patients with malignancy and febrile neutropenia, the most common organisms were* Streptococcus viridans*, Gram negative bacilli,* Staphylococcus epidermidis*,* Candida* sp., and* Salmonella* sp. [13].

The standard management of patients with febrile neutropenia includes cultures, hospitalization and close observation, and intravenous broad-spectrum antibiotics [14]. However, these patients comprise a heterogeneous group and possess different risks of developing severe infections and complications [15]. Models such as those developed by Talcott et al. [16] and the Multinational Association of Supportive Care in Cancer (MASCC) attempted to aid practitioners in classifying patients into low- and high-risk groups [17–19]. Using these models, poor prognostic variables identified in Western studies included a MASCC score <21, an Eastern Cooperative Oncology Group (ECOG) performance status score ≥2, chronic bronchitis and heart failure, a monocyte count <200 mm^3^, and stress hyperglycemia, among others [20]. In Korea, prognostic indicators included hypotension, previous and invasive fungal infection, recovery from neutropenia, median days to fever, pneumonia, and total febrile days [21, 22].

There is presently no such study in the Philippines. Being a third-world country with limited health resources, the study aims to provide Filipino data on prognostic factors associated with adverse outcomes and mortality and thereby recognize patients that need more intensive treatment and monitoring.

## 2. Materials and Methods

This retrospective study involved adult febrile neutropenia patients, regardless of cause, admitted to the Philippine General Hospital from January 2010 to October 2014. The inclusion criteria consisted of patients >19 years old with a suspected or documented risk factor for febrile neutropenia (hematologic malignancy, bone marrow failure state, or solid-organ tumor after myelosuppressive chemotherapy) who developed fever coexistent with or during the neutropenic episode; patients with existing severe infection prior to the onset of neutropenia were excluded. Patients were stratified into the “complicated group” if they developed severe outcomes, defined by any of the following: hypotension (systolic blood pressure < 90 mmHg and/or diastolic blood pressure < 60 mmHg), respiratory failure (arterial oxygen pressure < 60 mmHg on room air or need for mechanical ventilation), congestive heart failure, uncontrolled arrhythmia, hepatic or renal failure requiring treatment, severe bleeding requiring transfusion, altered sensorium, intensive care unit admission, and death; and they were stratified into the “noncomplicated group” if they did not manifest any severe outcome. Patients who left prior to recommended discharge were assumed to have developed severe outcomes and were categorized under the complicated group.

Clinical, laboratory, and microbiologic data were expressed as frequencies and percentages. Stepwise logistic regression with backward selection strategy was employed on specific variables; the significance of the main effects of the different independent variables on severe outcomes was determined by univariate analysis to establish the strength of association of each independent variable and outcome variable. Univariate test of any variable resulting in a *p* value ≤ 0.25 was considered a candidate for the multivariable model. Since some of the variables did not reach significance level *p* value ≤ 0.25 in the univariate analysis, a model that included significant variables was constructed. The variables included in the model were then subjected to multivariate analysis.

## 3. Results

### 3.1. Patient Characteristics

115 febrile episodes in 102 neutropenic patients were documented. Of these, 58 patients (50.4%) were classified under the complicated group and 57 (49.6%) fell under the noncomplicated group. There was no significant difference in the mean age and sex ratio of the patients between both groups. The overall mortality rate was 19.1%.

Almost half of the sample population had leukemia (*n* = 56, 48.7%) as the primary underlying disease, followed by solid-organ tumors (*n* = 27, 23.45%). In addition, 21 patients (18.26%) did not have a primary hematologic disorder or malignancy as the underlying disease; these included systemic lupus erythematosus (SLE), liver cirrhosis, HIV/AIDS, and drug-induced agranulocytosis. Majority of patients (*n* = 84, 73.04%) had no comorbidities. In those with comorbidities, cardiovascular disease (*n* = 21, 18.26%) had the highest frequency.

More patients in the complicated group did not receive treatment and/or had relapse of their underlying disease (*n* = 26, 44.83%). This nontreatment or relapse status significantly correlated with poor outcomes in the univariate analysis (OR 2.28; 95% CI, 1.04–4.98; *p* = 0.040). Similarly, prolonged duration of fever >7 days prior to admission (*n* = 16, 27.6%), nonrecovery from neutropenia (*n* = 41, 70.7%), and prolonged duration of neutropenia >7 days during admission (*n* = 31, 53.4%) were also seen more in the complicated group. However, only the last two variables (OR 2.17; 95% CI, 1.01–4.68; *p* = 0.048; OR 3.24; 95% CI, 1.16–9.01; *p* = 0.024, resp.) were significantly associated with poor outcomes in the univariate analysis. In contrast, more patients in the noncomplicated group received G-CSF (*n* = 23, 40.3%) but this did not reach statistical significance in the univariate analysis. The baseline characteristics of the study patients are summarized in Table 1 while the results of the univariate analysis for clinical variables are shown in Table 4.

### 3.2. Laboratory Characteristics

More subjects in the complicated group were observed to have ANC counts <100 on admission (*n* = 19, 32.8%) as well as nadir ANC counts <100 during the entire admission (*n* = 36, 62.1%). Likewise, more patients in this group had hemoglobin levels ≤ 80 g/L, platelet counts ≤ 50,000/*μ*L, peak BUN >20 mg/dL, peak creatinine >177 *μ*mol/L, nadir bicarbonate ≤ 21 mmol/L, nadir albumin ≤ 30 g/L, and peak AST and ALT levels > 40 IU/L. Among these factors, however, only severe thrombocytopenia showed significant prognostic correlation with poor outcomes in the univariate analysis (OR 3.45; 95% CI, 1.52–7.84; *p* = 0.003). The laboratory parameters of the study patients are summarized in Table 2 while the results of the univariate analysis are included in Table 4.

### 3.3. Microbiologic Characteristics

Of the 115 febrile episodes included in the study, 79 (68.7%) were clinically defined infections (CDI), 32 (27.83%) were microbiologically defined infections (MDI), and four (3.48%) had fever of unknown origin (FUO). A greater number of patients in the complicated group had MDI (*n* = 20, 34.5%). The most common site of infection was the respiratory tract (*n* = 58, 50.43%) in both groups, followed by the genitourinary tract (*n* = 13, 22.4%) in the complicated group and the oral cavity (*n* = 15, 26.3%) in the noncomplicated group. The overall bacteremia rate was 13.04%, but this did not show statistical significance in the univariate analysis.

*Pseudomonas aeruginosa* (*n* = 9, 7.83%) was the most commonly isolated organism, followed by* Escherichia coli* (*n* = 7, 6.09%). As a whole, Gram negative bacteria comprised the predominant isolate in the complicated group. Isolation of a pathogen in cultures, however, was not significantly associated with poor outcomes in the univariate analysis. In contrast, complete antibiotic therapy significantly predicted better outcomes in the univariate analysis (OR 0.26; 95% CI, 0.12–0.57; *p* = 0.001) with Piperacillin-Tazobactam being the most commonly administered antibiotic (*n* = 57, 49.57%).

The microbiologic profiles of the study patients are summarized in Table 3 while the results of the univariate analysis are displayed in Table 4.

### 3.4. Multivariate Analysis

Significant variables in the univariate analysis resulting in a *p* value ≤ 0.25 were considered candidates for the multivariable model. Since some variables did not reach significance level *p* value ≤ 0.25 in the univariate analysis, a model that included significant variables was constructed. Results of the multivariate analysis showed prolonged fever >7 days prior to admission (OR 2.43; 95% CI, 0.77–7.74) being associated with a significant risk for poorer outcomes. In the same manner, isolation of a known pathogen on cultures (OR 2.69; 95% CI, 1.04–6.98) and profound ANC < 100 during admission (OR 1.96; 95% CI, 0.75–5.12) were also found to be significant predictors of poor outcome. The factors that significantly correlated with better outcome, on the other hand, were G-CSF use (OR 0.31; 95% CI, 0.11–0.85) and completeness of antibiotic therapy (OR 0.26; 95% CI, 0.10–0.67). The results of the multivariate analysis are shown in Table 5.

## 4. Discussion

This is the first Philippine study dealing with prognostic variables in patients with febrile neutropenia. Our results generally show that febrile neutropenia confers a significant morbidity and mortality rate: 58 patients (50.4%) developed severe outcomes, with 22 (19.1%) of these eventually succumbing. This is consistent with the 5–21.5% mortality rate documented in the literature [3–5]. Leukemia was found to be the most common primary underlying disease, also consistent with the findings of earlier studies [3]. The nature of hematologic malignancies, plus the intensity of myelosuppressive treatment, predisposes these patients to a more rapid development of neutropenia and consequent infection, as compared to patients with solid tumors [19, 22].

Of the different clinical variables examined, prolonged fever >7 days prior to admission and G-CSF use both showed prognostic importance in the multivariate analysis. In contrast, our results did not consider the presence of comorbidities as significant indicators, as opposed to other studies [20–22]. This can be explained by the relatively small number of study patients with comorbid diseases. In terms of laboratory findings, multivariate analysis yielded a nadir ANC < 100 during admission as an indicator of adverse outcome. Since neutropenia is the main defect in host defense after chemotherapy, variables associated with neutropenia are closely related to infection susceptibility and are therefore likely to affect prognosis [21]. However, many of the study patients had incomplete workup (lacking, e.g., albumin, bicarbonate, and liver enzyme levels) and no data on levels of promising prognostic markers (procalcitonin, erythrocyte sedimentation rate, and C-reactive protein) [23, 24], thereby precluding the use of these variables for logistic regression analysis.

The predominance of the respiratory tract as the most common site of infection among study patients, as well as the isolation of* Pseudomonas* and* E*.* coli* as the most frequently implicated organisms, conformed with the results of previous studies [3, 21, 22]. In particular, the predominance of Gram negative bacteria in the complicated group affirms the reemergence of these pathogens as a result of more intensive chemotherapy regimens and the decreased use of quinolone prophylaxis [25].

A unique microbiologic profile observed in the study was the presence of tuberculosis in two cases, which was not observed in the literature. This can be explained by the unusually high prevalence of the disease in the country. Furthermore, no viral infection was documented in the study. As the main defect in host immunity after chemotherapy is in the innate rather than adaptive immunity, viral infections following chemotherapy do not seem to be common. The rarity of fungal infections, too, can be explained by the difficulty in confirming fungal infections as the condition of febrile neutropenic patients seldom permits invasive diagnostic procedures [21].

The study was limited by a small sample size and involvement of only one tertiary medical center and hence may have led to underestimation or overestimation of projections. It was also conducted in a resource-limited setting and was thereby constrained by the unavailability of full workup and ideal treatment in many patients, precluding these from further analyses. Finally, the data might not have been completely independent because several febrile episodes were assessed in the same patients. Thus, the presence of confounding factors, despite the use of an appropriate statistical model, cannot be fully excluded in multivariate analysis. This is a common difficulty encountered in retrospective studies [22].

## 5. Conclusion and Recommendations

Adult Filipino febrile neutropenia patients with prolonged fever >7 days prior to admission, known pathogen on cultures, and nadir ANC < 100 during admission were at significant risk of developing worse outcomes, whereas those with G-CSF use and complete antibiotic therapy were significantly associated with better outcomes. These variables might help identify patients with an increased risk of developing complications, thereby needing more intensive treatment and monitoring. The completeness of antibiotic therapy cannot be overemphasized and could lead to improved drug procurement policies for indigent patients. We recommend, however, bigger studies to further validate and strengthen our study findings.

## Figures and Tables

**Table 1:** Baseline clinical characteristics of febrile neutropenia cases.

Clinical parameter	Outcome frequency (%)	
	Complicated group *n* = 58 (50.4)	Noncomplicated group *n* = 57 (49.6)
Age		
Mean (SD)	41.24 (15.99)	40 (11.89)
Median	40.5	42
Range	19–71	19–61
Sex		
Male	23 (39.7)	24 (42.1)
Female	35 (60.3)	33 (57.9)
Primary underlying disease		
Leukemia	29 (50)	27 (47.4)
Lymphoma (all sites)	1 (1.7)	1 (1.8)
Solid-organ tumor	9 (15.5)	18 (31.6)
Bone marrow failure state	6 (10.3)	3 (5.2)
Others	13 (22.4)	8 (14.0)
Comorbid		
None	41 (70.7)	43 (7.5)
Cardiovascular disease	8 (13.8)	13 (22.8)
Pulmonary disease	1 (1.7)	0
Liver disease	0	1 (1.8)
Renal disease	3 (5.2)	0
Diabetes mellitus	2 (3.4)	0
Others	3 (5.2)	0
Outcome		
Discharged improved	32 (60.3)	57 (100.0)
Expired	22 (37.9)	0
Left against advice	4 (6.9)	0
Status of treatment		
Treated/on treatment	32 (55.2)	40 (70.2)
Remission	0	2 (3.5)
Relapse	3 (5.2)	3 (5.3)
Not on treatment	23 (39.7)	12 (21)
Type of treatment		
Chemotherapy	26 (44.8)	28 (49.1)
Combination therapy	6 (10.3)	17 (29.8)
None	26 (44.8)	12 (21.1)
G-CSF use		
Yes	16 (27.6)	23 (40.3)
No	42 (72.4)	34 (59.7)
Duration of fever prior to admission		
≤7 days	42 (72.4)	51 (89.5)
>7 days	16 (27.6)	6 (10.5)
Duration of neutropenia during admission		
≤7 days	27 (46.6)	35 (61.4)
>7 days	31 (53.4)	22 (39.6)
Recovery from neutropenia		
Yes	17 (29.3)	27 (47.4)
No	41 (70.7)	30 (52.6)

**Table 2:** Laboratory parameters of febrile neutropenia cases.

Laboratory parameter	Outcome frequency (%)	
	Complicated group	Noncomplicated group
ANC on admission (mm^−3^)		
≤100	19 (32.8)	13 (22.8)
>100	39 (67.2)	44 (77.2)
Nadir ANC during admission (mm^−3^)		
≤100	36 (62.1)	26 (45.6)
>100	22 (37.9)	31 (54.4)
Nadir hemoglobin (g/L)		
>80	18 (31)	23 (40.4)
≤80	40 (69)	34 (59.6)
Nadir platelet count (*μ*L^−3^)		
≤50,000	46 (79.3)	30 (52.6)
>50,000	12 (20.7)	27 (47.4)
Peak serum BUN (mg/dL)		
>20	3 (5.2)	0
≤20	35 (60.3)	29 (50.9)
No data	20 (34.5)	28 (49.1)
Peak serum creatinine (*μ*mol/L)		
>177	9 (15.5)	2 (3.5)
≤177	45 (81.8)	47 (82.5)
No data	4 (6.9)	8 (14)
Nadir serum bicarbonate (mmol/L)		
≤21	15 (25.9)	0
>21	5 (8.6)	5 (8.8)
No data	38 (65.5)	52 (91.2)
Nadir serum albumin (g/L)		
≤30	33 (56.9)	20 (35.1)
>30	10 (17.2)	6 (10.5)
No data	15 (25.9)	31 (54.4)
Peak serum AST (IU/L)		
>40	21 (36.2)	17 (29.8)
≤40	17 (29.3)	17 (29.8)
No data	20 (34.5)	23 (40.4)
Peak serum ALT (IU/L)		
>40	22 (37.9)	18 (31.6)
≤40	18 (31)	16 (28.1)
No data	18 (31)	23 (40.3)

**Table 3:** Microbiologic profiles of febrile neutropenia cases.

Microbiologic parameter	Outcome frequency (%)	
	Complicated group	Noncomplicated group
Infection type		
MDI	20 (34.5)	12 (21.1)
CDI	36 (62.1)	43 (75.4)
FUO	2 (3.4)	2 (3.5)
Site of infection		
Oral cavity	2 (3.4)	15 (26.3)
Respiratory tract	33 (56.9)	25 (43.9)
GI tract/intra-abdominal	4 (6.9)	2 (3.5)
Genitourinary tract	13 (22.4)	6 (10.5)
Skin and soft tissue	4 (6.9)	6 (10.5)
Unknown	1 (1.7)	0
Others	1 (1.7)	3 (5.6)
Bacteremia		
Yes	7 (12.1)	8 (14)
No	51 (87.9)	49 (86)
Isolated organism		
Gram positive bacteria, non-MDRO	3 (12)	0
Gram positive bacteria, MDRO	3 (12)	1 (6.7)
Gram negative bacteria, non-MDRO	8 (32)	4 (26.7)
Gram negative bacteria, MDRO	7 (28)	4 (26.7)
Fungus	4 (16)	4 (26.7)
TB	0	2 (13.3)
Antibiotic use		
Complete	21 (36.2)	39 (68.4)
Incomplete	37 (64.8)	18 (31.2)

^*∗*^MDRO: multi-drug-resistant organism.

**Table 4:** Prognostic factors related to severe outcomes based on univariate analysis.

Variable	OR (95% CI)	*p* value
Age	1.00 (0.98–1.03)	0.635
Sex		
Male		
Female	1.11 (0.53–2.33)	0.789
Primary underlying disease		
Hematologic disease	1.37 (0.65–2.87)	0.405
Others		
Status of treatment		
Treated		
Not treated	2.28 (1.04–4.98)	0.040
G-CSF use		
Yes	0.56 (0.26–1.23)	0.150
No		
Comorbid		
Yes	1.27 (0.56–2.91)	0.566
No		
Duration of fever prior to admission		
≤7 days		
>7 days	3.24 (1.16–9.01)	0.024
ANC on admission (mm^−3^)		
≤100	1.65 (0.72–3.77)	0.236
>100		
Nadir ANC during admission (mm^−3^)		
≤100	1.95 (0.93–4.10)	0.078
>100		
Duration of neutropenia during admission		
≤7 days		
>7 days	1.83 (0.87–3.84)	0.112
Recovery from neutropenia		
Yes		
No	2.17 (1.01–4.68)	0.048
Nadir hemoglobin (g/L)		
≤80	1.50 (0.70–3.24)	0.298
>80		
Nadir platelet count (*μ*L^−3^)		
≤50,000	3.45 (1.52–7.84)	0.003
>50,000		
Peak serum creatinine (*μ*mol/L)		
≤177		
>177	4.70 (0.96–22.95)	0.056
Bacteremia		
Yes	0.84 (0.28–2.49)	0.754
No		
Isolated organism		
Unknown		
Known	2.12 (0.97–4.65)	0.061
Antibiotic use		
Complete	0.26 (0.12–0.57)	0.001
Incomplete		

**Table 5:** Prognostic factors related to severe outcomes based on multivariate analysis.

Variable	OR (95% CI)	Standard error
G-CSF use		
Yes	0.31 (0.11–0.85)	0.16
No		
Duration of fever prior to admission		
≤7 days		
>7 days	2.44 (0.77–7.74)	1.44
Nadir ANC during admission (mm^−3^)		
≤100	1.96 (0.75–5.11)	0.96
>100		
Isolated organism		
Unknown		
Known	2.69 (1.04–6.98)	1.31
Antibiotic use		
Complete	0.26 (0.11–0.67)	0.12
Incomplete		
